# Rectal Foreign Bodies: Surgical Management and the Impact of Psychiatric Illness

**DOI:** 10.7759/cureus.26774

**Published:** 2022-07-12

**Authors:** Eoin O'Farrell, Ashim Chowdhury, Eva Maria Havelka, Ashish Shrestha

**Affiliations:** 1 General Surgery, William Harvey Hospital, Ashford, GBR

**Keywords:** surgical management, anorectal trauma, mental health, rectum, foreign body

## Abstract

Background

Entrapped rectal foreign bodies can be a challenge to manage and are being encountered by acute surgical teams with increasing frequency. The aims of our study were to (a) ascertain the population demographics of patients presenting with this problem in our local area of East Kent, (b) see if an association could be drawn between this presentation and proposed risk factors such as a psychiatric illness or socioeconomic deprivation, and (c) to review how foreign bodies are being removed in the East Kent Hospital University Foundation NHS Trust, United Kingdom and to highlight best practice with regards to this in line with the latest guidelines.

Methodology

Between 2017 and 2021, 32 cases of entrapped rectal foreign bodies were diagnosed and managed at our NHS Trust. Retrospective data taken from the theatre directory and electronic patient records were used to audit patient demographics, co-morbidities, the type of foreign body, and the extraction technique.

Results

The majority of patients (90%) were male (n = 29). The patients’ age ranged from 15 to 95 years, with a median age of 48 years. In total, 12 (37.5%) patients had a medical history of a psychiatric illness. The most common foreign bodies removed were sex toys or vibrators (n = 8) and roll-on deodorant bottles (n = 7). Kent Area B (n = 10) and Kent Area A (n = 9) were the areas with the highest number of cases. Twenty-two (68.8%) patients underwent examination under a general anaesthetic for removal, seven (21.8%) patients had the object manually removed without sedation, and three (9.4%) required a laparotomy with or without bowel resection.

Conclusions

Cases of an entrapped rectal foreign body in this local region typically involved male patients between 40 and 50 years old. A high proportion of this group had a history of a psychiatric illness supporting an association between this presentation and mental health. We have proposed some explanations for this association including the anal canal nervous system interplay with the “brain-gut axis.” Lower socioeconomic status and unemployment may also be risk factors for this surgical problem. A trans-anal approach for management is successful in the majority of cases; however, almost 10% of patients required emergency surgical management. We have highlighted best practice guidelines for the investigation and management of the entrapped rectal foreign body as part of our discussion.

## Introduction

The surgical dilemma of removing an entrapped rectal foreign body is being encountered more frequently by acute general surgical teams in the United Kingdom [1]. It is, however, by no means a new dilemma. The first description of an entrapped rectal foreign body in the medical literature dates back to the 16th century [2], while Smith reported the first case in 1939 [3] after he removed a shower hose from a patient’s rectum using a Kocher haemostat [4].

It is a presentation that is observed predominantly in men, who are roughly six times more likely than females to present to the emergency department (ED) with this problem [3]. The age at presentation according to previous literature is wide-ranging, from 20 years to over 90 years, with a median age of 44 years [2]. Incidence has been reported as 0.15 in 100,000 people, while another large surgical department reported removing one foreign body per month. However, it is difficult to truly account for incidence as patients typically only present to the ED when self-removal has failed [2,3]. The items used also range widely but the most common ones appear to be sexual devices and glass objects [2,3]. Other items mentioned in the literature include aerosol cans, light bulbs, broomsticks, vacuum components, construction materials such as tools and nails, as well as food items, usually fruits or vegetables [4].

The motivation behind the initial insertion of the item is usually for sexual arousal and pleasure; however, there are other reasons such as relief of constipation and concealment. Despite the historical taboo and shame culture surrounding anal practices, it is a common healthy consensual sexual practice among people of all genders and sexual orientations [4]. Accidental insertion is also a reason that is commonly reported. It may also, however, be a manifestation of a mental health problem or a result of an assault in which the item has been placed against the person’s will [5].

Mental illness has been linked to the insertion of rectal foreign bodies in the literature on numerous occasions, particularly in cases where the patient is unable to explain how the object entered the rectum. Routine psychiatry referrals have been suggested for all patients presenting with this problem, although clinical reasoning supporting this blanket referral is largely absent [4]. It seems that the intended benefit foreseen from these referrals would be the diagnosis of an underlying mental health condition and the prevention of a future recurrence. The impact of poverty on physical and mental well-being is well recognised, with lower incomes particularly predisposing individuals to greater risk of a mental health illness [6]. There is scarce discussion in the literature regarding an association between poverty or socioeconomic deprivation and the problem of an entrapped rectal foreign body.

Most people present to the ED within 24 hours [4] but there can be a reluctance to attend the hospital in some patients for a number of weeks, perhaps due to embarrassment [2]. While embarrassment is often stated as a reason for the delay, it is generally implied to be a patient problem, when there may also be clinician embarrassment contributing to the overall taboo leading to this delay [4]. Most foreign bodies can be removed safely without complications; however, there is a risk of damage to the bowel wall, including perforation, particularly during insertion and removal [4].

The aims of our study were to (a) ascertain the population demographics of patients presenting with this problem in our local area of East Kent, (b) see if an association could be drawn between this presentation and the proposed risk factors such as a psychiatric illness or socioeconomic deprivation, and (c) to review how foreign bodies are being removed in our NHS Trust, as well as to highlight best practice with regards to this in line with the latest guidelines.

This article was previously presented as an oral presentation at the 2022 Association of Surgeons of Great Britain and Ireland Annual Congress on May 3-5, 2022.

## Materials and methods

Our retrospective study encompassed a five-year period from 2017 to 2021. Three large district general hospitals in the East Kent Hospitals University Trust were included. Overall, there were 32 cases of an entrapped rectal foreign body analysed as part of this study. We excluded foreign bodies which were ingested, as well as those which were placed for a medical purpose, such as removal of an Endo-SPONGE. Patients were identified from the theatre directory, and their electronic records were used to collect data on patient demographics, co-morbidities, the type of foreign body, and the extraction technique.

The postcode address registered as the home address on the electronic records system and Census 2011 data, freely available online, was used as part of the socioeconomic analysis. Addresses registered as outside of Kent were not used in this analysis. The address data have been coded to ensure confidentially. Local authority areas in Kent are referred to as Area while postcodes within these local authorities have been numbered, such as Kent Area A Postcode 1.

Ethical approval was sought from and approved by the local clinical audit and improvement team at East Kent Hospitals University NHS Foundation Trust (Approval number: RN770541). All data used complied with GDPR principles. Identifiable data were stored on a password-protected NHS Trust computer and were only accessible to the research team members.

## Results

Of the 32 patients included in this study, 29 (90.6%) were male and three (9.4%) were female. The age at presentation ranged from 15 years to 95 years, with a median age of 48 years.

**Figure 1:**
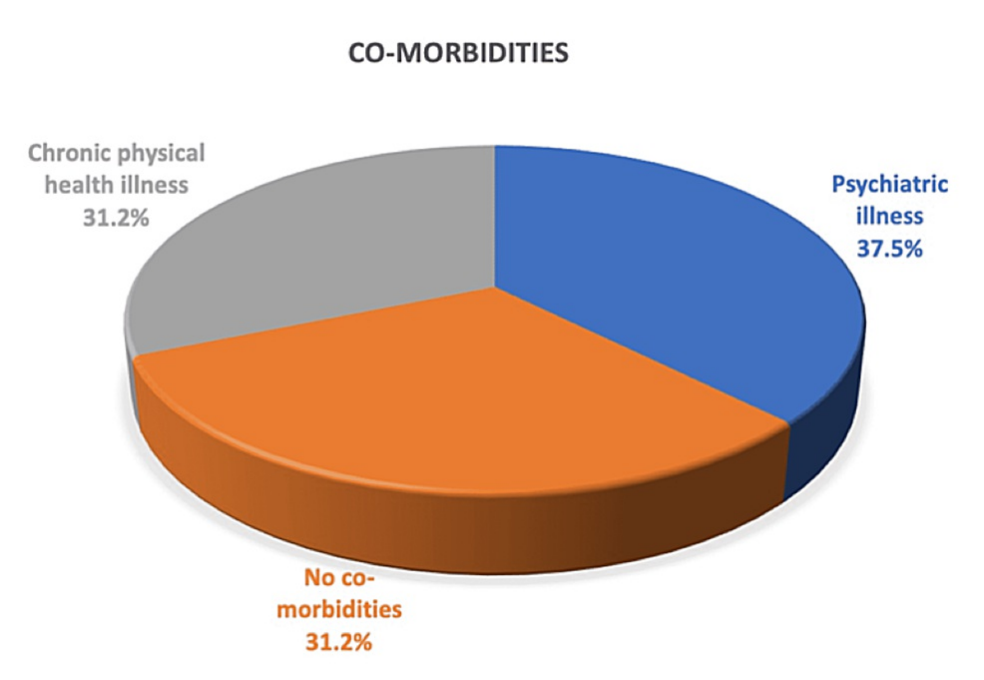
Breakdown of patient co-morbidities.

In total, 12 (37.5%) patients had been diagnosed in the past with a psychiatric illness, which included depression, anxiety, attention-deficit/hyperactivity disorder (ADHD), personality disorder, and psychotic disorder. A further 10 (31.2%) patients had a chronic physical health co-morbidity such as diabetes mellitus, Huntington’s disease, prostate cancer, long-term catheterisation, osteoarthritis, asthma, eczema, arterial hypertension, and erectile dysfunction. The remaining 10 (31.2%) patients had no significant mental or physical health problems in their medical history at presentation (Figure 1).

The marital status of 11 (34.3%) patients was not known at the time of data collection, 11 (34.3%) patients reported being single, and 10 (31.3%) patients were either married or with a long-term partner.

**Figure 2:**
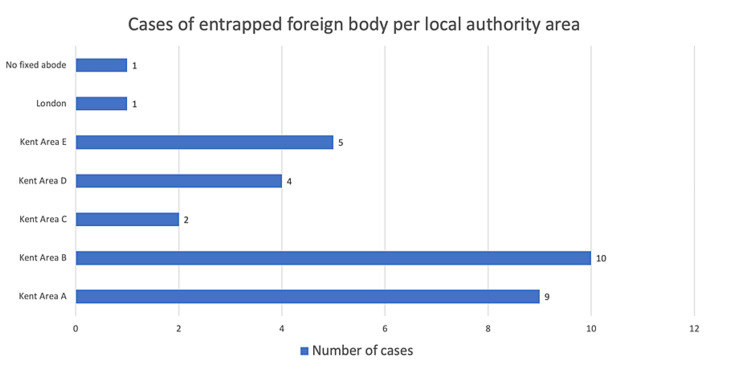
Cases of entrapped rectal foreign bodies as per the local authority area.

**Figure 3:**
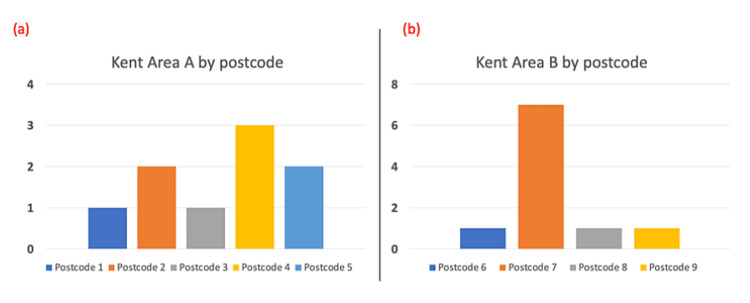
Cases of entrapped rectal foreign body as per postcode in the (a) Kent Area A and (b) Kent Area B local authority areas.

The local authority areas with the highest number of cases were Kent Area B and Kent Area A (Figure 2), where there were ten and nine cases, respectively. Within the local authority area of Kent Area A, the cases were relatively evenly distributed between five different postcodes, while within the local authority area of Kent Area B, one postcode, Postcode 7, contributed the significant majority, i.e., seven of ten cases (Figure 3).

A review of Census 2011 data identified that Kent Area A had a population of 166,800 with an unemployment rate of 3.0% and ranked eighth out of 12 local authority areas in Kent with regards to deprivation. Kent Area B had a population of 141,500 with a 5.7% unemployment rate and was ranked the most deprived area in Kent.

**Figure 4:**
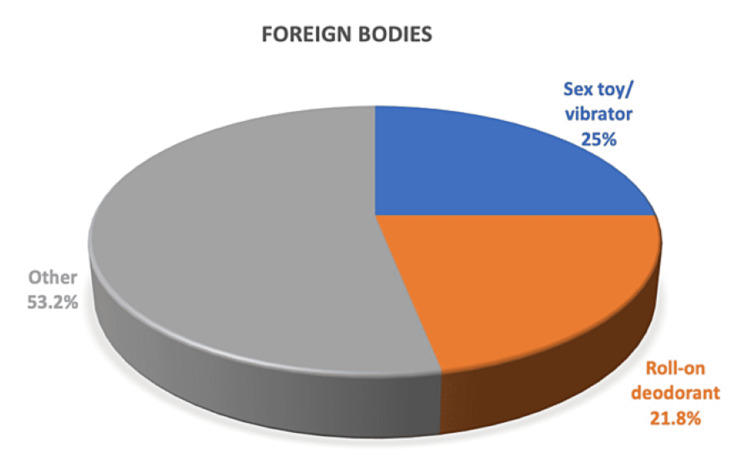
Types of entrapped rectal foreign body removed in this study group.

The most common items used (Figure 4) were sex toys/vibrators intended for sexual pleasure (n = 8, 25%), as well as roll-on deodorant bottles (n = 7, 21.8%). Other items included a wine glass, a hand torch, a plastic cream tube, a plastic bottle, a perfume bottle, a pepper grinder, a glass candle holder, an air-freshener canister, bathroom sealant applicator, a deodorant can, a bingo marker, a tennis ball, an electric toothbrush, a bottle cap, narcotics, an apple, and a cucumber.

**Figure 5:**
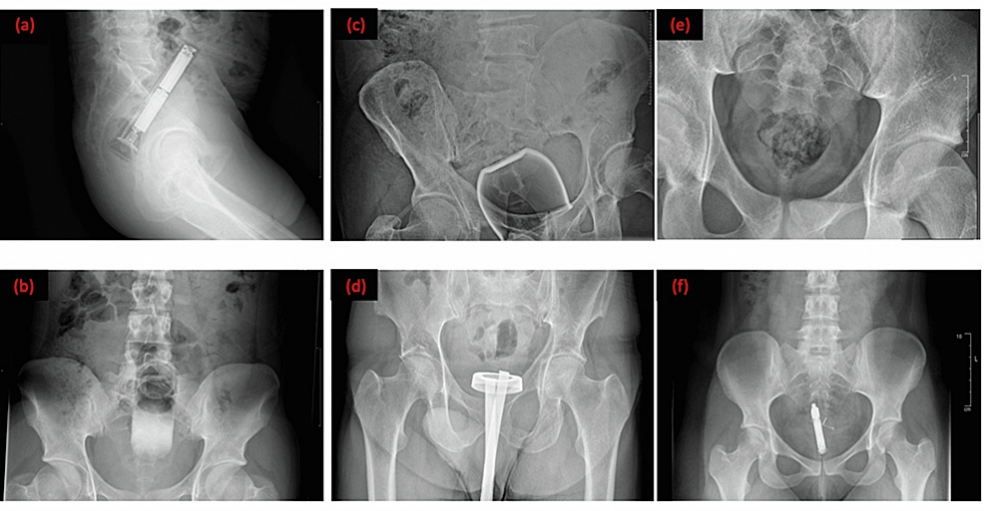
Radiological images of study cases on presentation: (a) hand torch, (b) roll-on deodorant, (c) glass candle holder, (d) bathroom sealant applicator, (e) narcotics, and (f) sex toy/vibrator.

Radiological imaging, some of which are in Figure 5, was available for review at the time of data collection in 30 of the 32 patients.

**Figure 6:**
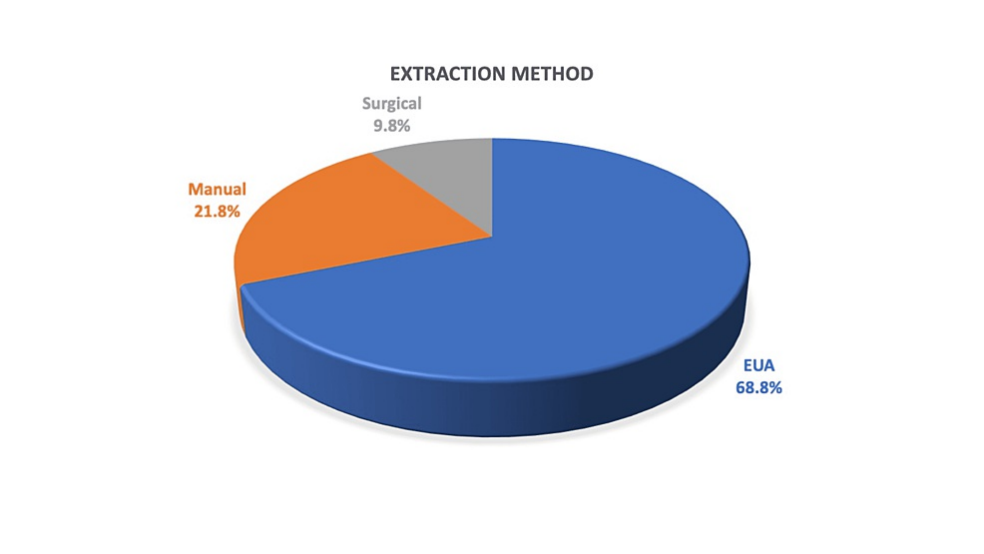
Extraction methods required in this study group to manage the entrapped rectal foreign body, including surgical management, examination under general anaesthesia, and manual evacuation without sedation in the emergency department. EUA = examination under anaesthesia

In total, 22 (68.8%) patients underwent examination under general anaesthetic (EUA) with the removal of foreign body and seven (21.8%) patients underwent manual evacuation in an emergency department. Three (9.4%) patients required a laparotomy for the removal of the entrapped foreign body (Figure 6).

## Discussion

Demographics

Concerning gender and age, our population was largely as expected according to previous literature with over 90% male and a median age of 48 years. The youngest patient in our group was 15 years old and the oldest was 95 years.

Symptoms

Many of the symptoms of an entrapped rectal foreign body are as expected, including bleeding from the back passage, abdominal pain, constipation, or faecal incontinence. However, one in five present with a different problem, and in these patients, a thorough history is essential to reveal the true reason they attended the hospital. Patients may camouflage their presenting complaint in this way due to embarrassment which is understandable, it can also be because the item has been placed involuntarily or under duress. In these circumstances, a clinician must have a high index of suspicion, particularly in vulnerable groups such as minors where safeguarding may be a priority. Patients who are experiencing a mental health problem are also an at-risk group who require a holistic approach to their care and potentially additional support from mental health services [2].

Mental health

Over one-third of the group (37.5%) had a medical history of a psychiatric illness. A large systematic review examining the global prevalence of common mental health disorders found that 29.2% of people had reported a mental health problem in their lifetime [7]. When compared with our study, the proportion of people in our group with a mental health issue is comparably higher than this, with a difference of 8.3%. In addition, another 31.2% had a significant chronic physical illness which has been shown to make them two to three times more likely to experience mental health problems than the general population [8]. This would suggest that it is likely the percentage of our group with a past or current psychiatric illness was even greater. There is little mentioned in the literature to explain why there seems to be this link between people with a psychiatric illness and the surgical dilemma of entrapped rectal foreign bodies.

The anal canal has a rich supply of nerve endings at the level of the skin which have a low threshold for stimulation and allow precise localisation and discrimination through activation of the somatosensory cortex. Findings from a study using functional magnetic resonance imaging indicated that non-painful anal stimulation also activates the limbic system which adds an emotional connection [9]. The penis and clitoris have a similar nervous system which explains its pleasurable effect and the role of anal stimulation in a healthy sexual relationship. Why then do we see a disproportionate number of people who have had a previous diagnosis of a psychiatric illness in our population? Through the pudendal and the vagus nerve, there is a well-established connection from the brain to the colon, commonly referred to as the “brain-gut axis.” Self-generated positive emotion via meditation has been shown to increase vagal tone and improve symptoms in those struggling with mood, anxiety, and chronic pain [10]. Perhaps then, through the use of a foreign body, stimulation of the anal canal has a similar effect on vagal tone, providing temporary relief from mental health symptoms.

Mental stress has also been shown to increase anal pressures via contraction of the internal anal sphincter through increased sympathetic nervous system activity [11]. Conversely, a relaxed state of mind is associated with reduced anal pressures in constipated patients. The study interpreting this data was doing so in patients with defecatory disorders, 48% of whom had a diagnosed psychiatric illness. The authors could not conclude whether a mental health problem caused the defecatory disorder or whether it was vice versa [11]. In relation to our study and owing to the bidirectional flow of information through the “brain-gut axis,” could manual relaxation of the internal anal sphincter through the placement of a foreign body induce a more relaxed state of mind?

Other researchers have found associations between an emotionally dysregulated state of mind and risky sexual behaviour, formed through maladaptive ways of responding to emotions, and it is thought therefore that risky sexual acts, such as inserting an item not intended for sexual pleasure into the rectum, may simply distract attention away from the intense emotion a person is feeling at the time [12].

It seems reasonable for clinicians to undertake a mental health assessment as part of their workup when treating patients who have presented with an entrapped rectal foreign body, with the use of brief assessment tools being suggested as practical [13]. The Patient Health Questionnaire Anxiety-Depression Scale (PHQ-ADS) (Appendix A), which combines the PHQ-9 and Generalized Anxiety Disorder 7 scales as a composite measure of depression and anxiety, has been shown to be effective in jointly assessing two of the most common psychiatric conditions seen in the general population [14]. In addition to offering psychiatric support where appropriate, clinicians should place a focus on education around safe sexual practices, increasing access to interventions, such as sexual health clinics, as well as alcohol and drug-use support if these are concerns [13].

Socioeconomics

Interestingly, the postcode area (Postcode 7) with by far the highest number of presentations (n = 7) lies within Kent Area B which is the most deprived local authority area in Kent and has the highest level of unemployment [15]. This suggests that there is an association between poverty and the surgical problem of an entrapped rectal foreign body. Poverty is associated with increased psychological stress and is suggested to reduce a person’s “cognitive band width.” This can contribute to poor decision making which can impact their health through engagement in risky behaviours [6]. In contrast, when the case data is accumulated by the local authority area, the data did not look as convincing in relation to this. Kent Area B, the most deprived district, had 10 cases overall, while Kent Area A, the eighth most deprived district, had nine cases overall. This would suggest poverty and unemployment have no impact on presentations to the ED with entrapped rectal foreign bodies. There is, however, a larger population in Kent Area A, with a more even distribution of cases, while in Kent Area B, one postcode contributes by far the most cases in this study, suggesting perhaps there are socioeconomic inequalities within the Kent Area B district itself which may be contributing to this effect.

Investigations

The majority of patients in this group had radiological imaging available for review. Guidance regarding best practice in assessing a patient with an entrapped rectal foreign body suggests a full abdominal examination should be performed, specifically assessing the abdomen for evidence of peritonitis. The next step, prior to performing a per rectal examination, should be to perform lateral and anterolateral plain X-ray films of the chest, abdomen, and pelvis. These are essential in the assessment of the shape, size, and location of the object, as well as for assessing sharp edges which may cause an accidental injury to the clinician’s finger. The X-ray images may also show pneumoperitoneum, evidence of perforated bowel. Routine pre-operative blood tests are indicated in those in whom manual evacuation has failed or if bowel perforation is suspected. If bowel perforation is suspected and the patient is haemodynamically stable, a CT abdomen with contrast should be performed to further assess prior to operating. However, if the patient is haemodynamically compromised, it is not recommended to delay surgical intervention for further imaging [16].

Management

Removal of the foreign body is usually attempted first through the least invasive method; however, there are some circumstances where less invasive methods are not appropriate [17]. Generally, the foreign body is classified as low lying or high lying and is defined by its position according to the recto-sigmoid junction. In the absence of peritonitis, manual removal at the bedside or endoscopic removal while the patient is conscious can be attempted. Failure of these methods requires examination under general anaesthesia and sometimes a laparotomy. A trans-anal approach was usually successful in removing the entrapped foreign body in this study group; however, three (9.4%) patients did require a laparotomy. Different tools for assisting the trans-anal removal have been described, including obstetric forceps, Kocher clamps, and bone cutters [2]. A Foley catheter has been used to hook the item from behind after the inflation of the balloon when it is placed appropriately. An extraction bag has also been designed and used, especially for spherical items which are difficult to grasp [18]. Trauma to the bowel wall is an important potential complication of the trans-anal approach to remove a foreign body. This can range from mild ulceration to a full-thickness perforation; therefore, a flexible sigmoidoscopy to assess for damage is considered standard of practice following removal. Patients should also be observed after the procedure for any signs of anorectal trauma or systemic signs of perforation. In the long term, there is a small risk of anal sphincter dysfunction due to local damage [2,16].

In the presence of peritonitis or haemodynamic compromise, the American Association for the Surgery of Trauma Organ Injury Scale [19] can be used to decide on surgical management, and there should be no delay in taking the patient to theatre for an emergency laparotomy if appropriate [16]. One patient in this group presented with localised peritonitis after he had voluntarily placed a glass object in his rectum which had broken into multiple pieces, perforating his bowel wall. He required an emergency Hartmann’s procedure which took over four hours to perform and in which 6 L of wash out was used due to faecal contamination of the abdominal cavity. He is currently on the waiting list for reversal of his temporary stoma. A second patient presented with perianal pain and rectal bleeding. He had a CT abdomen that revealed a foreign object in the rectum. Follow-up history regarding this finding revealed that the patient had fallen on a glass object 20 days previously. He was also managed with an open laparotomy and a sigmoid colectomy. In our third patient, a lower midline laparotomy was performed, and the item was pushed out rectally without needing to make an incision or resect a portion of the bowel. This patient required a suture to repair a small perforation in the bowel wall.

Overall, only seven (21.8%) patients had the rectal foreign body successfully removed at the bedside while conscious, meaning almost eight in ten patients required a fully staffed theatre for removal. With the average cost of one hour in theatre estimated at £1,200, this surgical problem has significant monetary implications for the Trust in addition to taking up valuable time in a busy emergency theatre [20]. Clinicians should follow best practice to fully assess the foreign object and remove through less invasive methods when possible. We recommend following the World Society of Emergency Surgery and the American Association for the Surgery of Trauma (guidelines [16] (Appendix B) in relation to the management of entrapped rectal foreign bodies.

Limitations

There are some limitations to our study which should be noted. Although the size of the patient population compares well with the literature, it is objectively a small sample size, and, therefore, the strength of analysis and conclusions are contained by this. As this was a retrospective study, the quality and range of data available for the collection were dependent on good documentation. Our time period also covers a changeover from paper documentation to electronic records, and, therefore, the quantity and quality of data available for patients seen during the era of electronic records were superior. Endoscopy services are in very high demand in our Trust; therefore, it was not considered for this patient cohort in terms of management options. We would aim to consider this option more in future as per the guidelines we have suggested.

## Conclusions

The demographics of the patient cohort in our local area are consistent with that found in the literature. Our data suggest that patients with a psychiatric illness are at a greater risk of requiring surgical management for an entrapped rectal foreign body. We have highlighted numerous potential explanations as to why it is a problem which more frequently presents in this particular group such as interplay with the parasympathetic nervous system and the subsequent effect on mood. We have also highlighted how clinicians should provide a holistic approach to the care of patients, particularly in this group. Poor socioeconomic status has potentially impacted our patient group, although the data can be interpreted differently depending on how it is viewed. We have examined our approach to the investigation and management of the entrapped rectal foreign body, and have made several suggestions, including the use of a mental health screening tool as well as best practice guidelines for the surgical management of this issue.

While we have shed light on several areas that have not been discussed in the literature to a great extent previously, there is still much discussion required around this surgical dilemma. A prospective study that also collected qualitative data from patients exploring their reasons for insertion of the rectal foreign body, their mental state if appropriate, and the circumstances of how the item became entrapped is suggested as a means of further understanding this surgical problem. The outcomes of such a study may provide clinicians with evidence-based advice for patients which could potentially help to avoid a life-changing surgery.

## Figures and Tables

**Figure 7:**
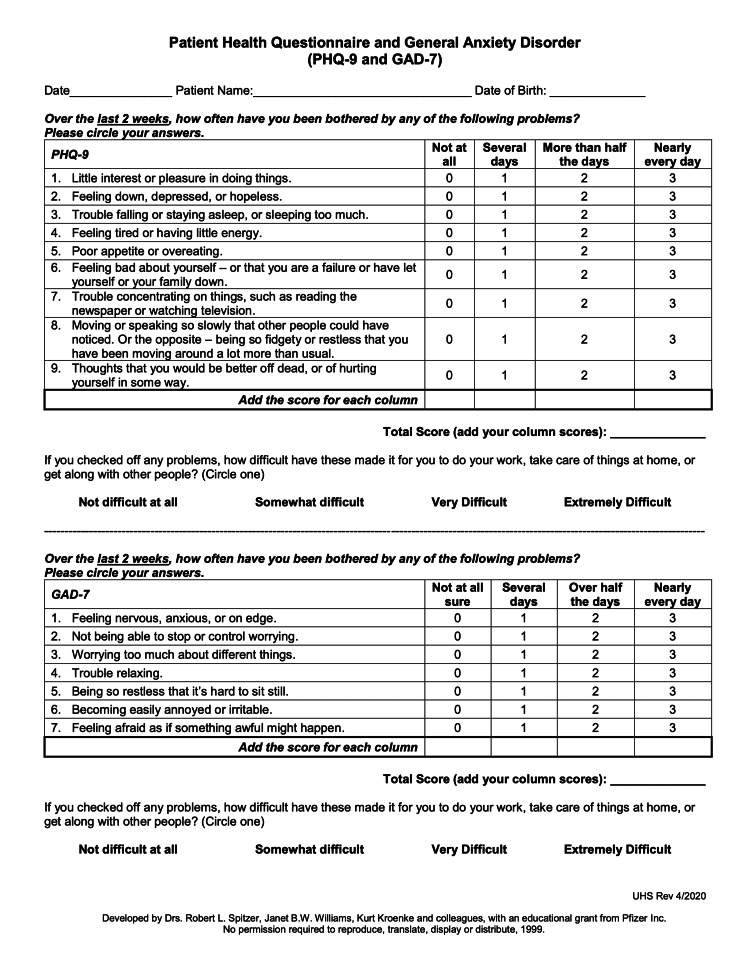
The Patient Health Questionnaire Anxiety-Depression Scale (PHQ-ADS).

## Appendices

Appendix A: The Patient Health Questionnaire Anxiety-Depression Scale (PHQ-ADS)

Appendix B: Recommendations as per Anorectal emergencies: WSES-AAST guidelines for the management of a retained anorectal foreign body

Retained anorectal foreign bodies

6A - In patients with a suspected retained anorectal foreign body, what is the role of clinical examination and biochemical investigations?

In patients with suspected retained anorectal foreign body, we suggest to collect a focused medical history and to perform a complete physical examination (weak recommendation based on low-quality evidence, 2C).

In patients with suspected retained anorectal foreign body, we suggest to perform a digital rectal examination after the acquisition of an abdomen X-ray, whenever possible, to prevent accidental injury to the surgeon from sharp objects (weak recommendation based on low-quality evidence, 2C).

In patients with suspected retained anorectal foreign body and no signs of bowel perforation, we suggest against routinely requesting laboratory tests (weak recommendation based on very low-quality evidence, 2D).

In patients with suspected retained anorectal foreign body and no signs of bowel perforation, we suggest to request routine preoperative blood tests only in case manual extraction fails/is not feasible (weak recommendation based on very low-quality evidence, 2D).

In patients with suspected retained anorectal foreign body and coexisting suspected bowel perforation, we suggest to request complete blood count and the dosage of serum creatinine, and inflammatory markers (e.g., C-reactive protein, procalcitonin, and lactates) to assess the status of the patient prior to surgery (weak recommendation based on low-quality evidence, 2C).

6B - In patients with a suspected retained anorectal foreign body, which are the appropriate imaging investigations?

In patients with suspected retained anorectal foreign body, we recommend lateral and anteroposterior plain X-ray film of the chest, abdomen, and pelvis to identify the foreign body position and determine its shape, size, and location and the possible presence of pneumoperitoneum (strong recommendation based on moderate-quality evidence, 1B).

In haemodynamically stable patients with suspected retained anorectal foreign body and a suspected perforation, we recommend a contrast-enhanced CT scan of the abdomen (strong recommendation based on moderate-quality evidence, 1B).

In patients with retained anorectal foreign body and hemodynamic instability, we suggest against delaying surgical treatment to perform imaging investigations (weak recommendation based on low-quality evidence, 2C).

6C - In patients with a retained anorectal foreign body, what is the most appropriate interventional approach (manual extraction vs endoscopy vs surgery)?

In patients with low-lying retained anorectal foreign body without sign of perforation, we suggest an attempt of bedside extraction as the first-line therapy (weak recommendation based on low-quality evidence, 2C).

No recommendation can be made regarding the superiority of one trans-anal extraction technique over the others in the case of retained anorectal foreign body, based on the available literature.

In patients with retained anorectal foreign body and failure of bedside extraction, we suggest pudendal nerve block, spinal anaesthesia, intravenous conscious sedation, or general anaesthesia to improve chances of trans-anal retrieval (weak recommendation based on low-quality evidence, 2C).

In patients with retained high-lying anorectal foreign body (above recto-sigmoid junction), we suggest an attempt of endoscopic extraction as the first-line therapy (weak recommendation based on low-quality evidence, 2C).

In patients with retained anorectal foreign body and suspect of drug concealment, we suggest against any manoeuver that can disrupt the drug package, including endoscopic retrieval (weak recommendation based on low-quality evidence, 2C).

In patients with retained anorectal foreign body, we suggest to perform a proctoscopy or flexible sigmoidoscopy after foreign body removal, to evaluate bowel wall status (weak recommendation based on low-quality evidence, 2C).

In patients with retained anorectal foreign body and signs and hemodynamic instability or perforation, we recommend against trans-anal extraction (strong recommendation based on low-quality evidence, 1C).

6D - In patients with a retained anorectal foreign body, what are the indications for surgical treatment and what is the appropriate timing for surgery?

In patients with retained anorectal foreign body and no signs of perforation, we suggest a surgical approach in case of failure of trans-anal extraction (weak recommendation based on low-quality evidence, 2C).

In patients with retained anorectal foreign body and no signs of perforation, we suggest a “step-up” surgical approach, starting with downward milking and proceeding to colotomy only when milking/trans-anal extraction fails (weak recommendation based on low-quality evidence, 2C).

In patients with retained anorectal foreign body and no signs of perforation, we suggest a laparoscopic approach if skills and instrumentation are available (weak recommendation based on low-quality evidence, 2C).

In patients with retained anorectal foreign body and bowel perforation with limited peritoneal contamination, we suggest primary suture only in case of small and recent perforation and if the colonic tissues appear healthy and well vascularised, and an approximation of perforation edges could be performed without tension (weak recommendation based on low-quality evidence, 2C).

In patients with retained anorectal foreign body and bowel perforation, clinically stable and without risk factors for anastomotic leakage, when a primary suture is not feasible, we suggest resection with primary anastomosis with or without a diverting stoma (weak recommendation based on low-quality evidence, 2C).

In critically ill patients with retained anorectal foreign body and bowel perforation, or in selected patients with extensive peritoneal contamination and risk factors for anastomotic leakage, we suggest to perform a Hartmann’s procedure (weak recommendation based on low-quality evidence, 2C).

In patients with retained anorectal foreign body and hemodynamic instability, we recommend an emergent laparotomy and a damage control surgery approach (strong recommendation based on moderate-quality evidence, 1B).

6E - In patients with a retained anorectal foreign body, is there a role for antibiotic therapy?

In patients with retained anorectal foreign body, we suggest against the routine use of antimicrobial therapy (weak recommendation based on low-quality evidence, 2C).

In patients with retained anorectal foreign body and signs of hemodynamic instability or perforation, we recommend broad-spectrum antibiotic therapy according to the WSES guidelines on intra-abdominal infections (strong recommendation based on moderate-quality evidence, 1B).
